# Differential impact of unilateral stroke on the bihemispheric motor cortex representation of the jaw and tongue muscles in young and aged rats

**DOI:** 10.3389/fneur.2024.1332916

**Published:** 2024-03-20

**Authors:** Miranda J. Cullins, Nadine P. Connor

**Affiliations:** ^1^Department of Surgery, University of Wisconsin-Madison, Madison, WI, United States; ^2^Department of Communication Sciences and Disorders, University of Wisconsin-Madison, Madison, WI, United States

**Keywords:** stroke, tongue, dysphagia, age, motor cortex, hemisphere, lateralization, corticobulbar

## Abstract

**Introduction:**

Dysphagia commonly occurs after stroke, yet the mechanisms of post-stroke corticobulbar plasticity are not well understood. While cortical activity associated with swallowing actions is bihemispheric, prior research has suggested that plasticity of the intact cortex may drive recovery of swallowing after unilateral stroke. Age may be an important factor as it is an independent predictor of dysphagia after stroke and neuroplasticity may be reduced with age. Based on previous clinical studies, we hypothesized that cranial muscle activating volumes may be expanded in the intact hemisphere and would contribute to swallowing function. We also hypothesized that older age would be associated with limited map expansion and reduced function. As such, our goal was to determine the impact of stroke and age on corticobulbar plasticity by examining the jaw and tongue muscle activating volumes within the bilateral sensorimotor cortices.

**Methods:**

Using the middle cerebral artery occlusion rat stroke model, intracortical microstimulation (ICMS) was used to map regions of sensorimotor cortex that activate tongue and jaw muscles in both hemispheres. Young adult (7 months) and aged (30 months) male F344 × BN rats underwent a stroke or sham-control surgery, followed by ICMS mapping 8 weeks later. Videofluoroscopy was used to assess oral-motor functions.

**Results:**

Increased activating volume of the sensorimotor cortex within the intact hemisphere was found only for jaw muscles, whereas significant stroke-related differences in tongue activating cortical volume were limited to the infarcted hemisphere. These stroke-related differences were correlated with infarct size, such that larger infarcts were associated with increased jaw representation in the intact hemisphere and decreased tongue representation in the infarcted hemisphere. We found that both age and stroke were independently associated with swallowing differences, weight loss, and increased corticomotor thresholds. Laterality of tongue and jaw representations in the sham-control group revealed variability between individuals and between muscles within individuals.

**Conclusion:**

Our findings suggest the role of the intact and infarcted hemispheres in the recovery of oral motor function may differ between the tongue and jaw muscles, which may have important implications for rehabilitation, especially hemisphere-specific neuromodulatory approaches. This study addressed the natural course of recovery after stroke; future work should expand to focus on rehabilitation.

## Introduction

Stroke frequently results in oral-motor deficits that lead to difficulty speaking and eating (1, 2). Dysplhagia (difficulty swallowing) is a particularly serious complication that occurs in up to 60% of people following stroke (3, 4). Post-stroke dysphagia is an independent predictor of poor outcomes and institutionalization (3, 5), and is associated with increased mortality rates, aspiration pneumonia, dehydration, and malnutrition (2, 3). Although many patients with post-stroke dysphagia recover some function with time, up to half may have swallowing problems that persist beyond 6 months (6). Thus, the recovery of oral-motor function after stroke is critical for both health and quality of life, yet clinical care is often limited to compensatory strategies due to a lack of evidence establishing any particular treatment (2, 7–9).

Improved treatment strategies for stroke-related dysphagia are needed, yet our ability to improve therapeutic options is limited by our incomplete understanding of the neurobiological mechanisms underlying recovery. Further, most of what is known about the mechanisms of post-stroke recovery are specific to the limbs; limited research has focused on the corticobulbar motor systems. Cranial motor nuclei controlling oral motor function are located in the brainstem and generally receive much more bilateral innervation than limb muscles (10–13). Thus, the neuroplastic mechanisms contributing to the recovery of swallowing function may differ substantially from recovery of sensorimotor limb function.

An important difference between the recovery of the cranial motor versus limb function may be which hemisphere of the brain is most critical for recovery—ipsilesional (infarcted) or contralesional (intact). It has been suggested that cortical plastic changes in the infarcted hemisphere are primarily important for hand and arm recovery (14, 15). In contrast, there is evidence that plasticity of the intact hemisphere drives the recovery of swallowing after stroke. For example, improvements in post stroke dysphagia were associated with increased cortical representation of the pharynx in the intact hemisphere, as measured by transcranial magnetic stimulation (TMS) (16). A meta-analysis also found that activation of the intact hemisphere by anodal transcranial direct current stimulation (tDCS) had a greater therapeutic effect on swallowing function than stimulation of either the infarcted hemisphere, or bilateral (17). Accordingly, a greater understanding of the bihemisphereic contributions to the recovery of oral motor function after stroke may be especially critical as many recent advances in post stroke therapy are focused on neuromodulation, often targeting the up or down regulation of activity in a particular hemisphere.

Age is also an important biological factor that is likely to impact the recovery of oral motor function after stroke, in that stroke most commonly occurs in aged individuals (18), age is an independent predictor of dysphagia after stroke (19), and neuroplasticity may be reduced with age (20–22). There are a number of aging effects specific to the motor cortex (23) which may impact how swallowing related motor representations respond after a stroke. Age has been associated with motor cortex thinning (24), hypoexcitability (25), and reduced plasticity of motor evoked potentials (26). Additionally, brain regions associated with mastication have been shown to have altered functional connectivity between the sensorimotor cortex and other brain regions in aged individuals (27). Accordingly, the aged oral motor system may be more susceptible to a decline in function after a stroke, and may impact the effectiveness of interventions. Thus, a better understanding of how age and stroke interact may lead to age-specific optimization of treatment strategies.

Animal models offer unique opportunities to address these mechanistic questions (28). Previously, we validated a rat model of post-stroke dysphagia using unilateral transient middle cerebral artery occlusion (MCAO) in 6 weeks-old male Sprague Dawley rats (29, 30). To address the impact of age on post-stroke oral motor function, we have adapted this model to young adult (7–9 months) and aged (30–32 months) Fisher 344-brown Norway rats, a standard rat model of aging (31).

We hypothesized that, after 8 weeks of recovery, unilateral stroke would be associated with expansion of the region of the sensorimotor cortex in the intact hemisphere that activates the jaw and tongue muscles. Further, we hypothesized that age would be associated with poorer oral motor function after stroke and a smaller oromotor activating volume in the intact hemisphere.

## Materials and methods

### Animals

All experiments were approved by the University of Wisconsin School of Medicine and Public Health Animal Care and Use Committee. The ARRIVE 2.0 guidelines were incorporated into the design and reporting of this work to promote experimental reproducibility (32).

Male Fisher 344 × Brown Norway rats, 40 aged (30 months old) and 29 young adult (7 months old), were obtained from the National Institute on Aging. Only males of this aging rat model were available at the time of this study. After 2 weeks of acclimation, rats were randomized into either stroke or sham surgery groups. In the aged group, 11 rats died, including 5 from the sham group (5 within 1 week of the stroke/sham surgery, 4 from 1–8 weeks after surgery, 2 during mapping). An additional 4 aged rats were excluded from analysis due to tumors in the neck, brain, or brainstem, which were likely to impact swallowing or motor maps. In the young adult group, 5 rats died within 1 week of stroke surgery. Final analyses, after exclusion of the aforementioned rats, included 25 aged (*N* = 11 sham, 14 stroke) and 24 young adult (*N* = 11 sham, 13 stroke) rats. Based on a previous study (33), the target sample size was 12 rats per group.

Videofluoroscopy of swallowing and mastication function was collected 7 weeks after surgery and intracortical microstimulation (ICMS) mapping occurred from 7–9 weeks after surgery. Spontaneous recovery in rats is considered to plateau by 4–6 weeks (15, 34, 35), thus data reflect a chronic functional status.

Final body weight was measured prior to ICMS mapping and normalized to baseline weight assessed prior to MCAO or sham surgery.

### Middle cerebral artery occlusion and sham surgeries

Transient occlusion of the left middle cerebral artery was performed using a minimally invasive approach developed by Hill and Nemoto (36). This approach offers advantages over the Longa method (37) as no arteries are severed and no sutures are left behind, which can cause adhesions and irritation in a region critical to oral motor function.

Rats were anesthetized with isoflurane and body temperature was maintained at 37°C using a heating pad during surgery and recovery. A midline neck incision exposed the junction of the left common carotid artery, external carotid artery, and internal carotid artery. Sutures and clips were used to control blood flow to allow for insertion of a monofilament (Doccol) through an opening pierced in the common carotid artery. Filament size was selected following manufacturer recommendations based on rat weight. The filament was carefully advanced to block the MCA for 25 min, then removed to restore blood flow. This occlusion duration was selected to optimize stroke survival in aged rats based on preliminary experiments.

Sham surgeries controlled for surgical effects by including all steps except MCA occlusion. While no cerebral damage occurs in the sham group, we will refer to the surgical side as the “infarcted hemisphere” in both the stroke and sham groups.

After recovering from surgery, rats were monitored daily and housed in cages with modified water bottles and food placement for ease of access. Signs of pain or discomfort including abnormal posture, decreased level of activity or interest, abnormal appearance of coat and skin, weight loss, age related tumor growth, and other behavioral signs of discomfort were addressed by a veterinarian and animals were removed from the study when recommended.

### ICMS mapping

The bilateral regions of the oral sensorimotor cortex that activate the jaw and tongue muscles were mapped using intracortical microstimulation based on previously published techniques (38–40). Rats were anesthetized for surgery with an IP injection of ketamine (70 mg/kg) and xylazine (9 mg/kg). A femoral IV line of ketamine was established to maintain anesthesia during mapping at 22.5 mg/kg/h, adjusted as needed to maintain the plane of anesthesia with the presence of a moderate toe-pinch response. Animals were placed in a stereotaxic apparatus, the cisterna magna was punctured to prevent edema, and the skull and dura were removed bilaterally over the motor cortex from 5 to 6 mm rostral and 2–3 mm caudal to bregma, and 6 mm lateral. Warm silicone oil was placed on the cortical surface.

**Figure 1:**
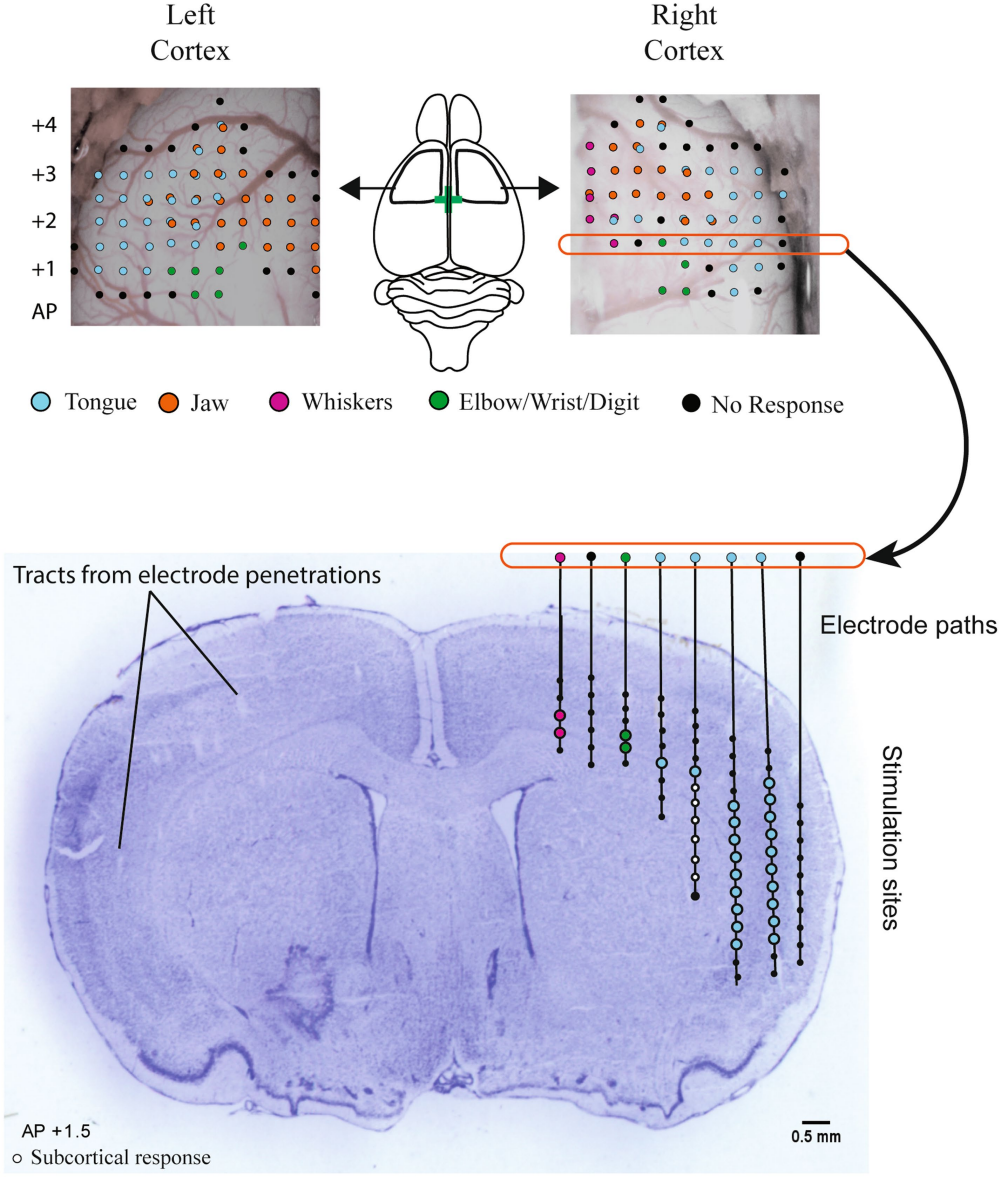
Intracortical microstimulation. Top panel: example maps of bilateral electrode penetration sites. Each site was stimulated at multiple depths and is colored to indicate all muscle group responses. Central diagram illustrates the approximate location of bilateral craniotomies with bregma indicated by a green cross. Stimulation occurred at each penetration site at 250 μm intervals starting at a depth of 1,200 μm below the cortical surface until responses disappeared, up to 5,000 μm. Stimulation sites for the right hemisphere at +1.5 AP are shown registered on a cresyl-violet stained coronal brain section (Bottom panel). Electrode penetration tracks can be visualized on the left hemisphere. The activated body part (tongue, jaw, etc.) at each stimulation site was recorded to determine the total cortical volume activating the tongue and jaw in each hemisphere, with the exclusion of sites determined to be subcortical (white dots) based on rat brain atlas registration.

A digital photo of each hemisphere was superimposed with a 250 μm grid in Adobe Illustrator. The grid was aligned with bregma and used as a guide for electrode placement. Electrode penetrations began at 1.0 mm AP and 3.0 mm ML from bregma and were spaced at 0.5 mm following grid lines and avoiding blood vessels. At each penetration site, stimulation was delivered starting at 1,250 μm from the surface and at 250 μm of increased microelectrode penetration depth until no more jaw or tongue ICMS-evoked movements could be detected, up to a depth of 5,000 μm (Figure 1). Stimulation consisted of thirteen 200 μs cathodal pulses delivered at 350 Hz from an electrically isolated stimulation circuit at a supramaximal stimulation current of 60 μA. Increased stimulation currents of up to 250 μA were necessary to elicit a motor response in some rats (41); sites that did not elicit a motor response at 250 μm were recorded as non-responsive. The procedure was continued until the entire jaw and tongue activating volumes were mapped. The investigator that performed mapping was blinded to the treatment group.

### ICMS and infarct volume analysis

**Figure 2:**
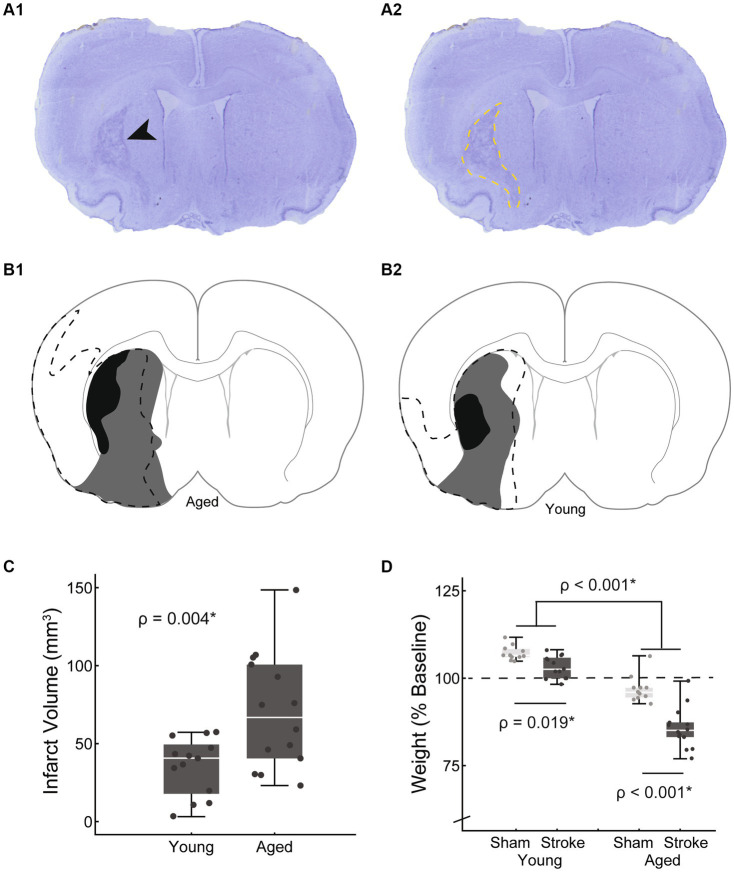
Cresyl violet staining was used to verify that an infarct occurred and to estimate the volume of the infarcted tissue. An example section is shown with a black arrow indicating the infarcted region **(A1)** which is outlined in **A2** (yellow dashed line). Representative example maps of infarct size variations at approximately AP + 1.2 from bregma are shown for aged **(B1)** and young adult rats **(B2)**. The smallest, largest, and median stroke volumes are represented (black, dashed line, and gray respectively). Infarct volumes were significantly larger in the aged group vs. young adult [**C**: young = 34.1 ± 18.7 mm^3^, aged = 70.3 ± 36.9 mm^3^, *t*(19.6) = −3.25, *p* = 0.004, mean ± SD]. Finals weights, normalized to baseline are shown by age and treatment group **(D)**. The dashed line at 100% represents no weight change with points above the line representing weight gain, and those below weight loss. Both age and stroke were associated with significant weight loss [age: *F*(1,45) = 141.6, *p* < 0.001; stroke: *F*(45,1) = 42.5, *p* < 0.01].

After mapping was complete, brains were frozen, sectioned by cryostat (Leica) at 50 μm, and stained with cresyl violet. Based on visible electrode penetration tracks and rat brain atlas registration (42), stimulation sites determined to be subcortical were removed from analysis (Figure 1). This generally corresponded to depths >2,500 μm and >2 mm medial of the lateral edge of the brain at 1.0 mm AP, > 3,000 μm deep and >2.5 mm from the lateral edge at 1.5 mm AP, and >3,000 μm and >3 mm from lateral edge at 2 mm AP. The volume of the sensorimotor cortex that activated the tongue and jaw muscles was calculated by multiplying the number of positive stimulation sites by 0.0625 mm^3^ (0.5 × 0.5 × 0.25). Sites that activated both the jaw and tongue were counted in both the jaw and tongue volumes. The same cresyl violet stained sections were used to determine infarct volume. Cresyl violet staining highlights the infarct core, which appears darker or as a dark border around extremely damaged tissue when used in rats that survive a week or more after occlusion (43). To determine the infarct volume, approximately 6–8 cresyl violet stained sections spanning the infarct were selected. Damaged areas were outlined (Figure 2A) in ImageJ (44) and the volume was calculated using the infarct areas and distances between sections.

### Tongue EMG

Bilateral bipolar hook-wire electrodes (Natus) were inserted ventrally into the middle of tongue to monitor EMG signals to confirm lingual muscle activation during ICMS. The EMG signal was recorded on a computer using the Muscle SpikerBox Pro and Spike Recorder software (Backyard Brains) sampled at 5 kHz per channel with a 60 Hz notch and 30–500 Hz bandpass filters. Jaw EMG was not used as jaw movement was more easily visualized.

### Videofluoroscopy

Videofluoroscopy was used to assess swallowing function and mastication 7 weeks after stroke and sham surgeries. Using an established vidoefluorographic swallowing study protocol (29, 45) a mixture of peanut butter and barium sulfate (Varibar Thin Honey) was placed on an elevated stage in the rat home cage and video of *ad libitum* feeding was collected at 30 frames per second on a C-Arm fluoroscopy machine (either continuous mode or pulsed at 30 pulses per second). Mean bolus area and jaw excursion were selected to assess tongue and jaw function respectively, based on their significant changes with stroke in a previous study (29). Images were analyzed by a single investigator blinded to treatment group.

**Figure 3:**
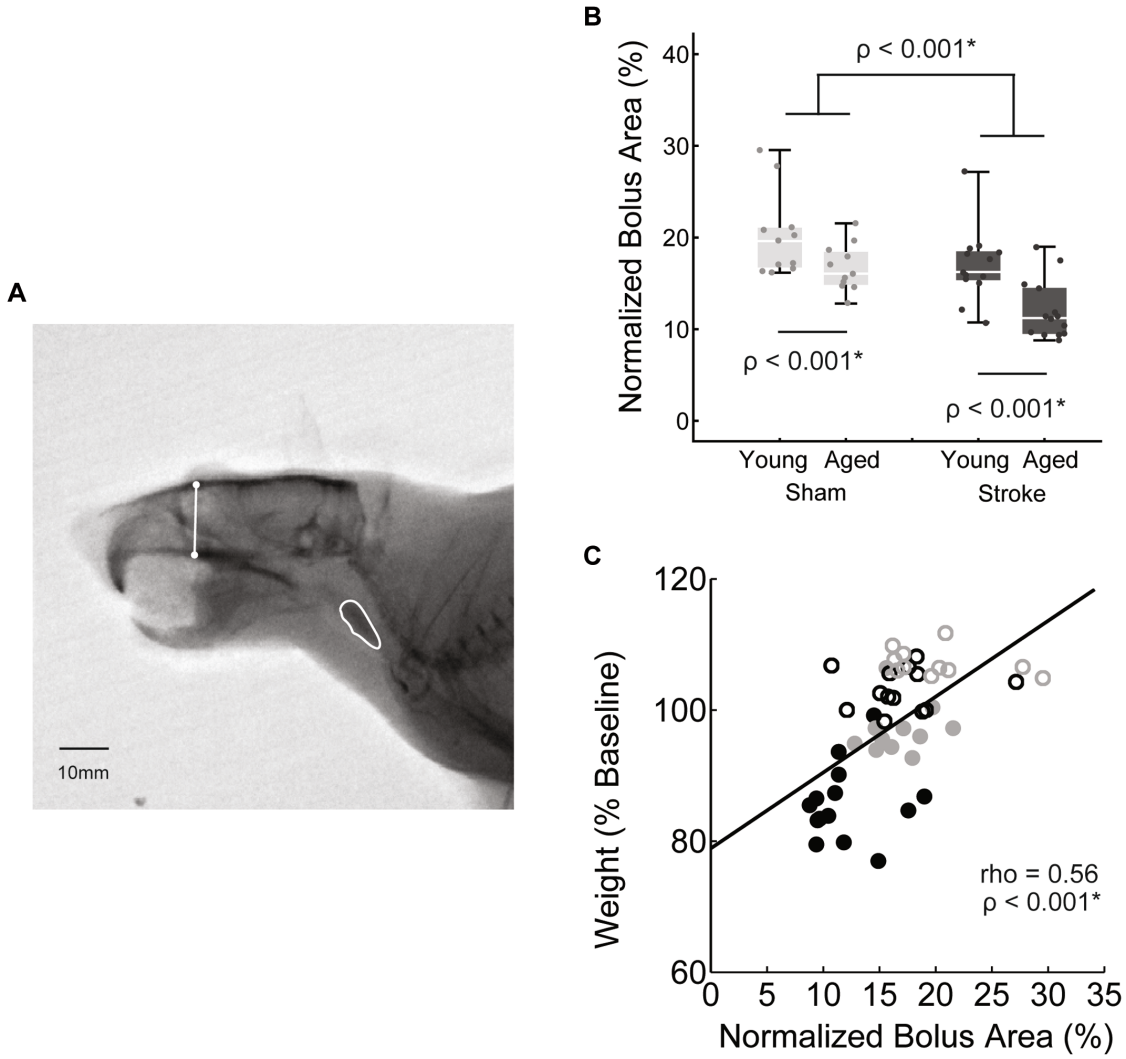
Swallowing bolus size reduced with stroke and age. **(A)** Bolus areas (outlined in white) were normalized to the square of a scull size based scalar (white line) to account for differences in rat body size. **(B)** Both stroke and age were associated with significantly smaller mean bolus area [*F*(45,1) = 16.2, *p* < 0.001; *F*(45,1) = 14.4, *p* < 0.001 respectively; young sham = 20.2 ± 4.6, young stroke = 17.0 ± 4.0, aged sham = 16.7 ± 2.6, aged stroke = 12.1 ± 3.2; mean ± SD %]. **(C)** Overall there was a moderate positive correlation between bolus area and normalized body weight, such that smaller mean boluses were associated with greater declines in body weight.

Bolus area was measured using ImageJ (44) to trace the bolus 1 frame before it entered the upper esophageal sphincter (approximately vertebrate C4). To account for swallow-to-swallow variability of bolus size, 15–20 swallows with a clear sagittal view were measured from each rat to determine individual mean bolus areas. To account for differences in rat size, especially between the young and old rats (mean weights of 411 ± 25 g and 504 ± 56 g respectively), bolus area was normalized. In human videofluoroscopy, the square of the distance between vertebrae’s C2–C4 has been used as a scalar when measuring areas (46). In the rat, C2–C4 distance was difficult to measure reliably, therefore we used the square of a previously established rat skull size measure, facial height (Figure 3) (47). Bolus area is reported as a percentage of facial height squared.

**Figure 4:**
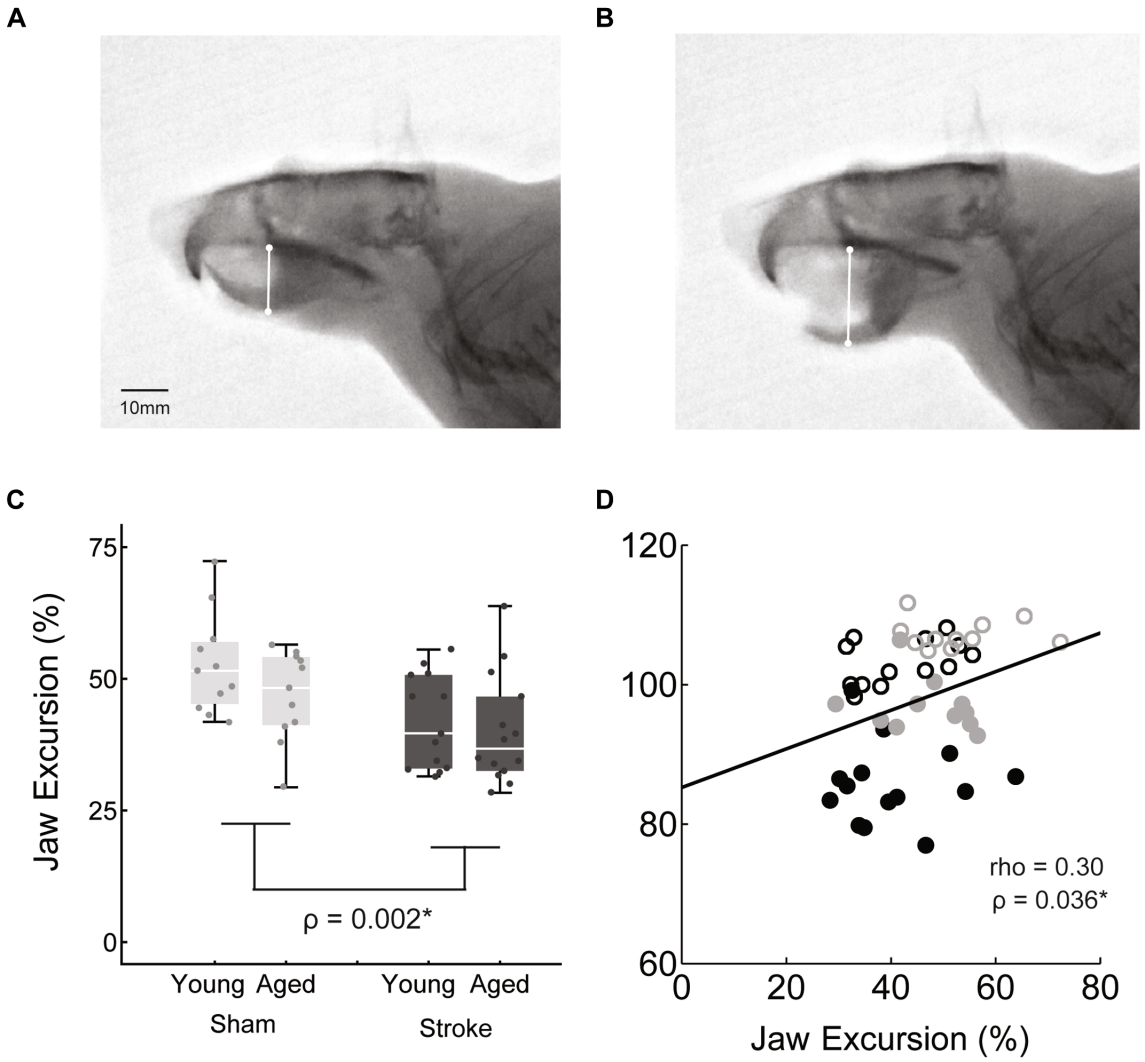
Jaw excursion during mastication was measured as the difference between the distance from the hard pallet to the mandible at jaws closed **(A)** and jaws opened **(B)**, normalized to the jaws closed distance. Jaw excursion distance was significantly reduced with stroke across age groups [**C**: stroke = 41.0 ± 9.5, sham = 49.8 ± 9.3, *F*(45,1) = 10.5, *p* = 0.002]. **(D)** The correlation between jaw excursion and weight was a weak positive.

Jaw excursion was determined by measuring the distance between the hard palate and the mandible when the jaw was closed and at the peak of opening (29). The difference between these two measurements was then normalized to the jaw closed measure, to account for differences in rat size (Figure 4).

### Statistics

ANOVA was used to compare bolus area, jaw excursion, and weight with age and treatment group as the between subject factors. Jaw and tongue activating cortical volumes were assessed by repeated measures MANOVA with hemisphere as the within-subject factor and the between subject factors of age and treatment group.

Independent samples *t*-tests were used to compare lateralization of jaw and tongue cortical representations. An independent samples t-test was also used to compare the infarct volume between age groups. Due to significant differences in variance between the groups, the *t*-value calculated using Satterthwaite’s method is reported.

The number of rats with elevated stimulation thresholds (>60 μA) were compared between groups by age and treatment group using Fisher’s exact test.

Pearson’s correlation was used to assess the relationships between infarct size and kinematic measures with jaw and tongue activating cortical volumes. Spearman’s rho was substituted when data were not normally distributed.

An alpha level of 0.05 was used for all statistical tests. All statistical results are derived from the complete dated set of 25 aged (*N* = 11 sham, 14 stroke) and 24 young adult (*N* = 11 sham, 13 stroke) rats.

## Results

### Infarct size

Representative example maps of infarct locations and size variations are shown in Figure 2. Infarcts were centered in the striatum and frequently extended into the cortex. One third of the strokes were subcortical with the rest a mix of cortical and subcortical. Infarct volumes were significantly larger in the aged vs. young adult rats [70.3 ± 36.9 mm^3^, 34.1 ± 18.7 mm^3^, *t*(19.6) = −3.25, *p* = 0.004, mean ± SD, Figure 2C]. To account for this confounding factor, a secondary analysis with infarct volume included as a covariate was run when directly comparing the young adult and aged stroke groups.

### Normalized weight

Final weights were normalized to baseline such that numbers over 100 represent weight gain and less than 100 weight loss. Both age and stroke were associated with reduced weight [main effect age: *F*(1,45) = 141.6, *p* < 0.001; main effect treatment: *F*(45,1) = 42.5, *p* < 0.01, Figure 2D]. Across treatment groups young rats maintained or slightly increased in weight (young adult sham = 107.2 ± 2.1, young adult stroke = 103.2 ± 3.2% baseline weight, M ± SD) and aged rats lost weight (aged sham = 96.9 ± 3.8%, aged stroke = 85.8 ± 5.8% baseline weight, M ± SD). This age-based result is as expected based on published growth curves (31); on average male F344 × BN rats gain weight through the first 25 months of life and weight declines after 30 months of life (31). Over the course of this 2 months study, the young adult group aged from 7 to 9 months, and the aged group aged from 30 to 32 months.

The interaction between age and treatment was significant [*F*(45,1) = 9.27, *p* = 0.004], with the aged stroke group having a greater weight difference than age-matched sham (young adult stroke vs. sham: −4.1%, aged stroke vs. sham: −11.2%). In both age groups, the stroke group had significantly lower normalized weight than the age-matched sham control group (young adult: *p* = 0.019, aged: *p* < 0.001). While the significantly larger infarcts in the aged stroke group is a confounding factor, this interaction remains significant when infarct volume is included as a covariate [*F*(44,1) = 5.8, *p* = 0.02].

### Videofluoroscopy kinematics

Videofluoroscopy was used to assess the impact of stroke and age on both swallowing and mastication. Bolus area, a functional swallowing measure, was normalized to skull size as described in the methods. Normalized bolus area was significantly smaller with both age [*F*(45,1) = 16.2, *p* < 0.001] and stroke [*F*(45,1) = 14.4, *p* < 0.001; young adult sham = 20.2 ± 4.6, young adult stroke = 17.0 ± 4.0, aged sham = 16.7 ± 2.6, aged stroke = 12.1 ± 3.2; mean ± SD %]. The interaction between age and treatment was not significant [*F*(45,1) = 0.46, *p* = 0.50].

Jaw excursion was significantly reduced in the stroke group [stroke = 41.0 ± 9.5, sham = 49.8 ± 9.3, M ± SD, *F*(45,1) = 10.5, *p* = 0.002], but there was no significant effect of age [*F*(45,1) = 0.1, *p* = 0.15] and no significant interaction between age and treatment [*F*(45,1) = 0.5, *p* = 0.46].

The relationship between these kinematic measures of oral motor function (jaw excusion, bolus area) and body weight was assessed via correlation analysis. Both measures were positively correlated with weight, such that reduced bolus area and jaw excursion were associated with reduced weight, however bolus area was moderately correlated (rho = 0.56, *p* < 0.001^*^), whereas jaw opening was only weakly correlated (rho = 0.3, *p* = 0.036^*^).

### ICMS—volume

**Figure 5:**
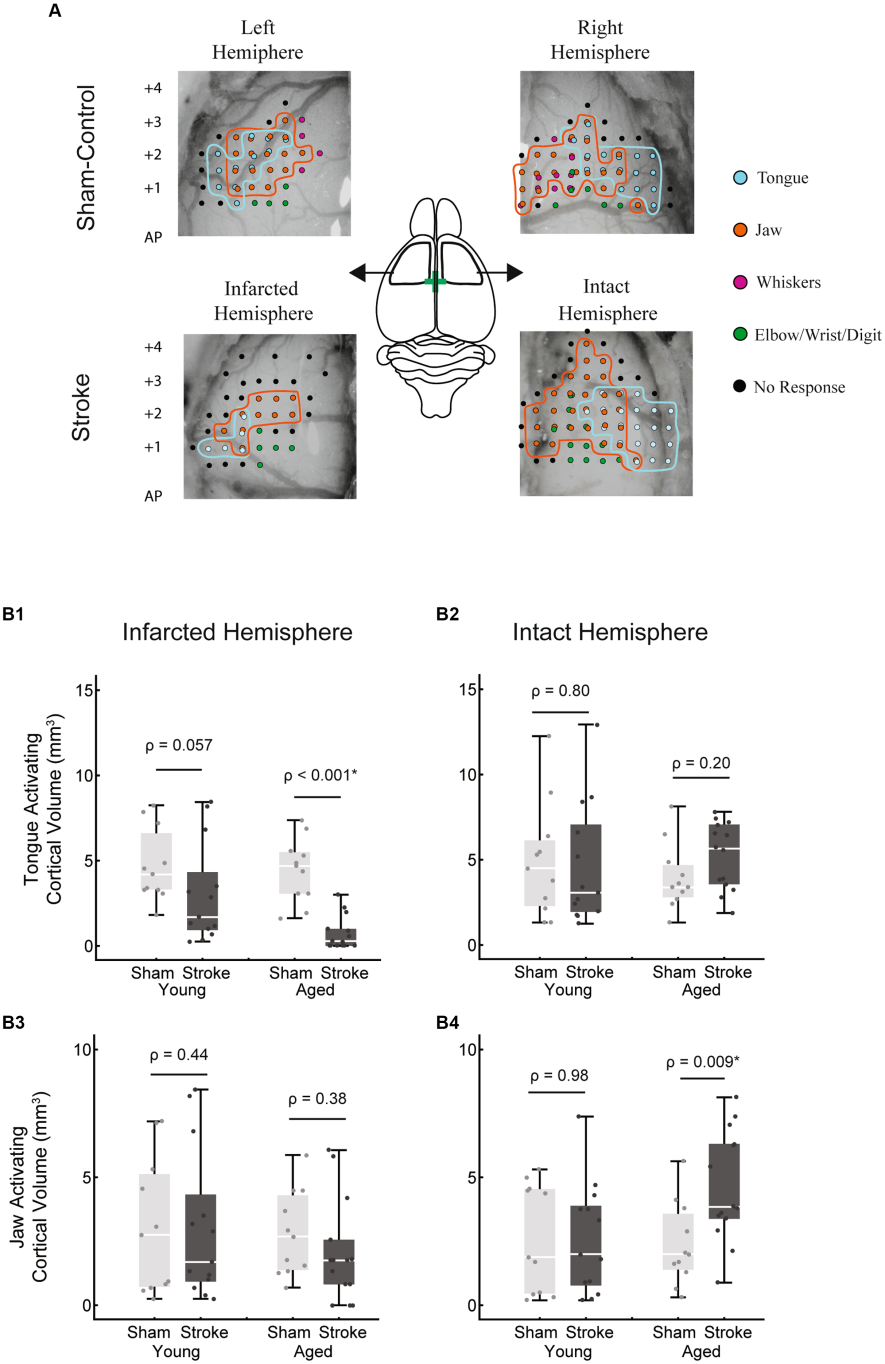
Tongue and jaw activating cortical volumes. **(A)** Representative examples of bilateral 2D cortical electrode penetration maps in stroke and sham-control rats. Each penetration site represents multiple stimulation depths collapsed onto a 2D map. Tongue activating sites are indicated in cyan and jaw activating sites in orange. **(B1)** The tongue activating cortical volume in the infarcted hemisphere was significantly reduced with stroke in the aged group (*p* < 0.001) but failed to reach significance in the young adult group (*p* = 0.057). Within the stroke group, the tongue activating volume in this hemisphere was smaller in the aged group, however this difference was not significant when corrected for differences in infarct size between age groups. **(B2)** In the intact hemisphere, no significant differences in tongue activating cortical volume occurred between groups. No significant differences in jaw activating volume occurred in the infarcted hemisphere **(B3)**. In the intact hemisphere, within the aged group, jaw activating volume was significantly larger in the stroke group **(B4)**. The jaw activating volume in the aged stroke group was larger than the young stroke group, however this difference was not significant when corrected for differences in infarct size.

Intracortical microstimulation was used to assess the bilateral cortical volumes that activated the tongue and jaw muscles when stimulated. Based on previous clinical studies we expanded cortical volumes in the intact hemisphere to contribute to swallowing recovery. There was a significant multivariate interaction between hemisphere, treatment (stroke), and age [*F*(44,2) = 5.2, *p* = 0.009; Figure 5].

In the infarcted hemisphere, the tongue activating volume was smaller in the stroke vs. sham group, however this difference was only significant within the aged group [Figure 5B1; age stroke = 0.73 ± 1.00 mm^3^, aged sham = 4.43 ± 1.87 mm^3^, mean ± SD; *F*(45,1) = 19.32, *p* < 0.001; young adult stroke = 3.04 ± 2.93 mm^3^, young adult sham = 4.71 ± 2.14 mm^3^, mean ± SD; *F*(45,1) = 3.82, *p* = 0.057].

Jaw activating volume in the infarcted hemisphere was not significantly different as a function of age or treatment. However, in the intact hemisphere, there was a significantly larger jaw activating region in the aged stroke vs. aged sham group [Figure 5B4; age stroke = 4.62 ± 2.15 mm^3^, aged sham = 2.37 ± 1.60 mm^3^, mean ± SD; *F*(45,1) = 7.51, *p* < 0.009].

Within the stroke group, age-related differences included a smaller tongue activating volume in the infarcted hemisphere (Figure 5B1; age stroke = 0.73 ± 1.00 mm^3^, young adult stroke = 3.04 ± 2.93 mm^3^) and a larger jaw activating volume in the intact hemisphere (Figure 5B4; age stroke = 4.62 ± 2.15 mm^3^, young adult stroke = 2.59 ± 2.16 mm^3^). However, these differences were not statistically significant when adjusted for differences in infarct volume between the age groups [*F*(24,1) = 2.25, *p* = 0.15; *F*(24,1) = 1.63, *p* = 0.22 respectively; evaluated at an adjusted mean infarct of 52.88 mm^3^].

### Lateralization

**Figure 6:**
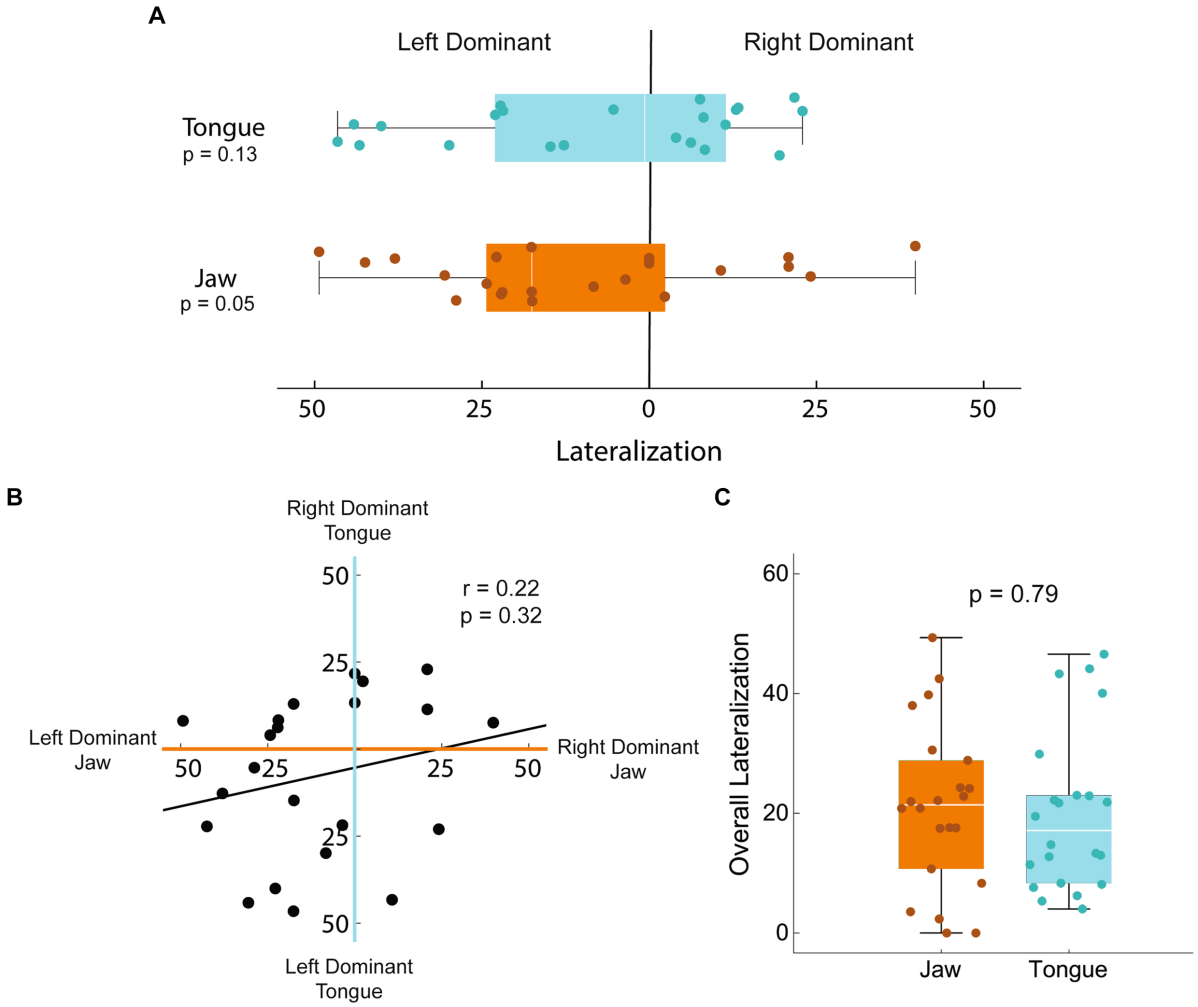
Lateralization of jaw and tongue activation in sham-controls. Lateralization of the jaw and tongue activating volumes was calculated as the difference between hemispheres normalized to the total bihemispheric representation. A value of 0 represents equal map sizes in each hemisphere; a 50% left dominant lateralization would have 75% of the total map volume in the left hemisphere and 25 in the right. **(A)** Lateralization was variable, with neither hemisphere statistically more commonly dominant in either tongue or jaw function (*T* = −2.07, *p* = 0.051; *T* = 3.25, *p* = 0.005 respectively). **(B)** Correlation between the laterality of the jaw and the tongue was negligible (Pearson’s *r* = 0.22, *p* = 0.32) such that the degree of dominance of one muscle in a given hemisphere does not predict the other. **(C)** The absolute degree of lateralization, independent of hemisphere, was not significantly different between the jaw and tongue (*T* = 0.26, *p* = 0.79).

To understand the potential impact of hemispheric dominance on our results, we calculated the degree of lateralization of jaw and tongue activation in the sham-control group (Figure 6). Lateralization was quantified by calculating the difference in muscle activating volume between the two hemispheres and normalizing by the total bihemispheric representation. There was a tendency for the jaw to be left hemisphere dominant, however this difference was not significant (*T* = −2.07, *p* = 0.051). For the tongue, rats were equally likely to be left vs. right hemisphere dominant, although larger degrees of lateralization occurred in the left hemisphere (*T* = 3.25, *p* = 0.005). Correlation between the laterality of the jaw and the tongue was negligible (Pearson’s *r* = 0.22, *p* = 0.32) such that the degree of dominance of one structure in a given hemisphere does not predict the other. The absolute degree of lateralization, independent of hemisphere, was not significantly different between the jaw and tongue (*T* = 0.26, *p* = 0.79). Lateralization analysis was limited to the sham-control group as the originally dominant hemisphere of each rat in the stroke group is not known; mapping was only carried out at the end of the study.

### Differential impact of infarct volume on the size of tongue and jaw activating cortical regions

**Figure 7:**
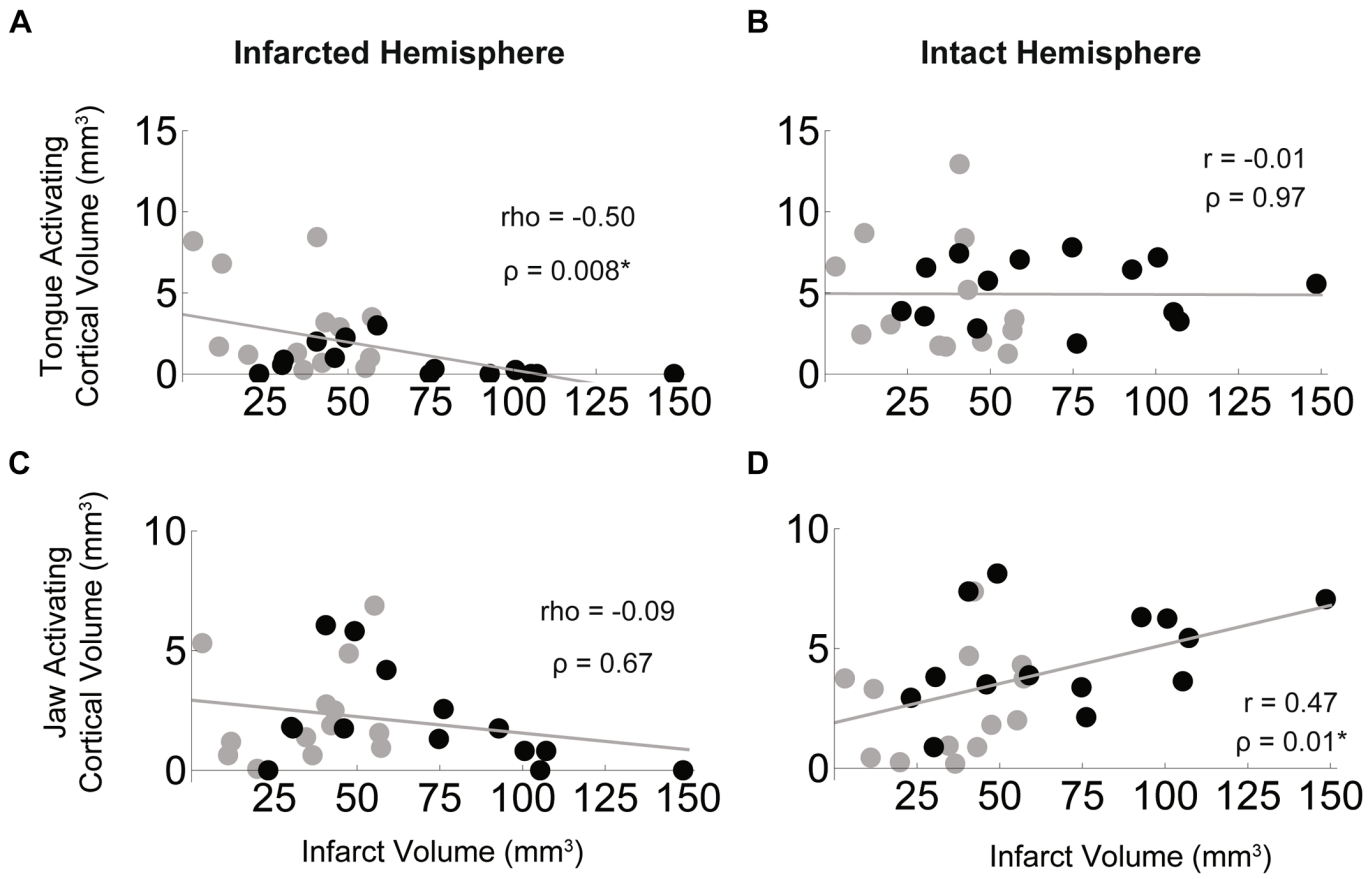
Bihemispheric impact of infarct size on tongue and jaw muscle activating cortical volumes. In the stroke group (black = aged, grey = young) larger infarcts were associated with smaller regions of cortex activating the tongue in the infarcted hemisphere **(A)** but infarct size was not correlated with the size of the tongue activating region of the intact hemisphere **(B)**. For the jaw, infarct size was not significantly correlated with size of the jaw activating region in the infarcted hemisphere **(C)**, but larger infarcts were associated with larger jaw activating regions in the intact hemisphere **(D)**.

We found that the size of the infarct was negatively correlated with the size of the tongue activating region in the infarcted hemisphere (rho = −0.50, *p* = 0.008), but was not significantly correlated with tongue activating volume of the intact hemisphere (Figures 7A,B). The opposite was found for jaw muscles in that the relationship between size of the infarct and jaw activating region in the infarcted hemisphere was not significant (Figures 7C,D), while jaw activating region of the intact hemisphere was moderately, significantly, and positively correlated with infarct size (*r* = 0.47, *p* = 0.01).

### Relationship between ICMS volumes and oral-motor functions

**Figure 8:**
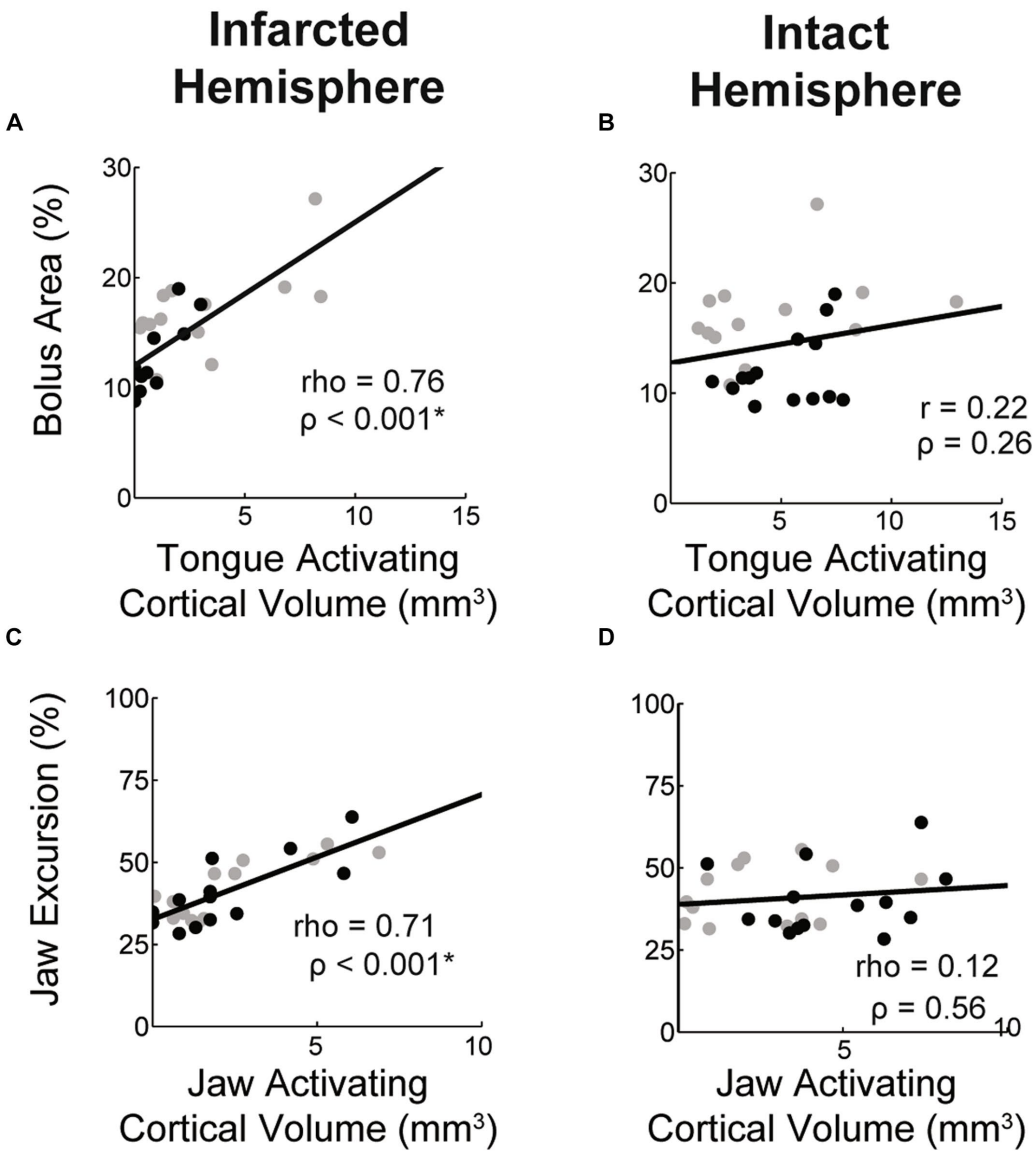
Correlations between muscle activating cortical volumes and function. Within the stroke group the volume of the cortex activating the tongue and jaw in the lesioned hemisphere was highly correlated with bolus area and jaw excursion, respectively (A,C). Values for aged (black) and young (grey) stroke groups are plotted separately. Correlation strengths between these functional measures and the jaw and tongue activating cortical volumes in the contralesional hemisphere were low (B,D).

We sought to determine the relationship between muscle activating cortical volumes in each hemisphere and related oral-motor functions. We found that jaw and tongue activating volumes in the infarcted hemisphere were significantly and highly correlated with jaw excursion and bolus area, respectively (Figures 8A,C). Jaw and tongue activating volumes in the intact hemisphere were not significantly correlated with oral-motor function (Figures 8B,D).

### ICMS—thresholds

We found that both stroke and age were associated with elevated stimulation thresholds such that current levels greater than 60 μA were required to initiate muscle movements (Table 1). In the sham group, all the young adult rats were fully mapped at 60 μA, while 36% of the aged rats had elevated stimulation thresholds. In the stroke group, 86% of the aged and 46% of the young adult rats required elevated stimulation to initiate movements. We found elevated thresholds occurred only in the lesioned hemisphere of the young stroke group, yet in the aged stroke group 2 of 12 also had increased thresholds in the intact hemisphere. Of the aged sham-control rats with elevated thresholds, 3 out of 4 were elevated in both hemispheres, suggesting that contralesional activation threshold increases may be associated with age rather than stroke. Specifically, the incidence of increased contralesional activation threshold was significantly increased with age (*p* = 0.028; Fisher’s exact test, one-sided), but was not significantly different with stroke (*p* = 0.40; Fisher’s exact test, one-sided).

**Table 1:** Stroke and age associated with elevated stimulation thresholds.

	Sham	Stroke	
Aged	36% (4/11)	86% (12/14)	*p* < 0.001^*^
Young	0% (0/11)	46% (6/13)	*p* < 0.001^*^
	*p* < 0.001^*^	*p* < 0.001^*^	

The number of rats with elevated stimulation movement thresholds (>60 μA) was significantly higher in the stroke vs. sham groups. This number was also significantly higher with age in both the stroke and sham groups. *p*-values were determined using Fisher’s exact test.

## Discussion

Our findings suggest that the role of the intact and infarcted hemispheres in the recovery of oral motor function may differ between the tongue and jaw muscles, which may have important implications for post stroke recovery of swallowing and speech. This was the first study to model post stroke dysphagia in aged (30–32 months) rats. Across treatment groups, age was associated with swallowing differences, weight loss, and increased stimulation thresholds of the motor cortex independent of stroke, however increased infarct size in the aged stroke group made it difficult to differentiate between the effects of age and stroke size within the stroke group.

### Hemispheric cortical plasticity of jaw and tongue motor maps

Previous studies of patients with post stroke dysphagia suggest that improved swallowing function is associated with increased cortical representation of the pharyngeal muscles in the intact hemisphere (16, 48). Therefore, we hypothesized that 8 weeks after unilateral stroke, the cortical map size of the jaw and tongue would increase in the intact hemisphere. Partially in line with this hypothesis, we found a significant increase in the size of the intact hemisphere motor map of the jaw but did not for the tongue (Figure 5).

Why might the corticomotor plasticity of the tongue differ from other swallowing muscle groups? The tongue muscles are unique from other cranial muscles in several ways, including their embryological origin, innervation, and range of movements (49). Most muscle groups involved in speech and swallowing, including the facial, jaw, laryngeal, and pharyngeal muscles are branchiomeric muscles, originating from the cranial mesoderm, while the tongue has a somitic origin similar to trunk and limb muscles (50–53). Most cranial motor neurons receive bilateral innervation from the corticobulbar pyramidal tract, yet the innervation of the tongue, while still bilateral, is considered to be primarily crossed (12, 13, 54, 55). The tongue is capable of a vast repertoire of unique movements and regional deformations due to its complex patterns of muscle fiber orientations and motor unit activation (49). Additionally, the tongue has unique mechanosensory structures and acuity comparable to fingertips (56, 57). These unique motor and sensory innervations and movement patterns may contribute to divergent recovery and rehabilitation plasticity.

Differential cortical plasticity of lingual function after stroke could have important clinical implications for the treatment of post stroke dysphagia and dysarthria. Neuromodulatory therapeutic strategies, which have shown some positive effects for post stroke dysphagia (17, 58), are often hemisphere specific and seek to enhance recovery by increasing plasticity through unilateral cortical activation or inhibition. Our results suggest that the ideal pattern of neuromodulatory intervention may differ between the tongue and other groups of swallowing muscles. It is important to stress that the results of the current study reflect a natural course of recovery without any therapeutic intervention. It is possible that clinically based interventions such as tongue exercise would drive a different pattern of cortical plasticity, and these mechanisms should be pursued in future studies.

A limitation of this study is that motor maps and oral motor function were not assessed at multiple time points. We found that lateralization of motor maps between the two hemispheres was highly individual among sham-control rats (Figure 6), thus knowing what the motor maps looked like in each individual prior to stroke would allow for a much more precise determination of the relationships between functional recovery and motor map plasticity in the acute and chronic phases. However, it was not possible to perform the mapping experiment used in this study pre-stroke and survive the rats as required by our research questions. Longitudinal studies of upper limp maps have been achieved using techniques such as optical imaging and implanted cranial windows (59). Similar to the upper limb, jaw motor maps are located on the dorsal surface of the brain and would be accessible using these techniques. However, the depth and laterality of much of the tongue activating cortical region (60) (Figure 1) may render it inaccessible to established chronic mapping techniques. There is some evidence that the expansion of motor maps can be transient, expanding during the development of a new skill, then retract once that movement pattern has matured (61). It is possible that peak expansion of oromotor maps occurred earlier in the post stroke timeline and had already consolidated at our 8 weeks mapping point. However, a longitudinal study of mouse forelimb motor and sensory map changes after stroke found changes persisted at 6–8 weeks post stroke (62).

Additional time points of functional assessments would allow us to differentiate between individual rats that maintained good function after the stroke, those that suffered a loss of function but exhibited substantial functional recovery, and those that lost function but experienced limited recovery. Quantifying the progress of functional recovery rather than just end point function may further clarify the variability in motor map sizes (16).

In this study we focused on the jaw and tongue muscles as activation of these muscles are easily visualized and have historically been included in studies mapping the rat sensorimotor cortex. Based on our differential results, similar studies of additional swallowing structures, such as the pharyngeal and laryngeal muscles, would be of great interest and allow for a more comprehensive picture of how the bihemispheric contributions to the recovery of swallowing and speech after unilateral stroke.

### Lateralization

Many studies have reported lateralization or hemispheric dominance of swallowing with mixed results (63). These discrepancies may be attributed to both individual differences in lateralization and differential lateralization of muscle groups and swallowing tasks within individuals. It is important to consider the impact of variable lateralization when interpreting unilateral stroke.

In our study, the degree of lateralization and hemispheric dominance in the sham-control group varied on an individual basis. For the jaw, a dominant left hemisphere was more common, but this was not significant. Further, laterality of the jaw and tongue were not significantly correlated (Figure 6), such that the dominant jaw hemisphere did not significantly predict laterality of tongue representation. Our results are in accordance with an earlier human study that mapped the cortical topography of the myohyoid, pharyngeal, and esophageal muscles. The representation of these swallowing muscles was frequently asymmetrical, and the dominant side varying not only between individuals, but between muscles within an individual (48).

The variability of lateralization of the jaw and tongue muscles may explain some of the variability between the stroke size and function seen in this rat stroke model (30, 64). A similarly sized infarct may have a much greater functional impact when it occurs in the dominant hemisphere rather than the non-dominant hemisphere. Establishing the dominant hemisphere in this model prior to stroke would allow for more detailed analyses of these relationships.

### Age

We sought to determine the impact of age on post-stroke oral motor function. We identified several relevant age-based differences independent of stroke: across treatment groups, age was associated with increased stimulation thresholds, weight loss, and smaller bolus areas when swallowing. These findings are consistent with the aging literature: reduced excitability of the motor cortex with age has been established via studies using transcranial magnetic stimulation (25), and weight loss is a common occurrence with advanced age that has been associated with functional decline (65, 66). Most clinical swallowing assessments use controlled bolus sizes, so the direct impact of age on bolus size is not well established, however several studies have reported age being associated with a higher frequency of segmented swallowing of large boluses (breaking a bolus into smaller boluses, also referred to as piecemeal or fractional swallowing) (67–69).

Stroke impacted each of these factors in a similar manner: reduced weight, bolus size, and cortical excitability were also associated with stroke in each age group. Because age and stroke impact these factors in similar ways, aged individuals may be at a disadvantage for recovering optimal oral motor function after stroke. For example, the age-related changes found in the present study may reflect a decline in the functional reserve of the oral motor systems. The term “functional reserve” describes the difference between maximum effort and the level of effort and biological resource recruitment used to accomplish a task (70). A decline in this reserve with age may limit the potential for recovery after a stroke, as fewer untapped resources are available to be recruited.

When comparing the impact of stroke between the age groups, significantly larger infarct volumes in the aged group were a confounding factor (Figure 2C). The difference in infarct size with age was not expected as previous reports have not found a consistent relationship between age and infarct size in male rodent stroke models (71). Future studies may benefit from matching infarct size between groups by lengthening the duration of MCA occlusion in the young adult group. After correcting for infarct size, we did find a significant interaction between age and stroke, such that aged stroke resulted in greater weight loss (Figure 2D). Weight loss after stroke is a common occurrence which may be multifactorial, yet is strongly associated with difficulty eating (72). Tracking food intake in future studies would allow for exploration of the relationships between functional swallowing changes, nutritional intake, and weight loss after stroke.

### Stimulation thresholds

Reduced cortical excitability has been found to occur both due to age (25) and stroke (73), which is in agreement with our finding that increased stimulation currents were required to drive a motor response in a subset of stroke and aged rats (Table 1).

Higher stimulation thresholds suggest greater recruitment of cortical neurons was required to elicit muscle activation. ICMS activates layer 5 pyramidal neurons of the motor cortex via both direct and transsynaptic activation, with higher stimulation currents increasing direct activation (74). Higher stimulation currents have also been shown to recruit a larger area of the cortex (75). Damage to the pyramidal tracts has also been found to increased cortical stimulation thresholds (76, 77). Thus, after stroke, the increased stimulation needed to drive a motor response in this study may reflect increased pyramidal neuron recruitment to overcome changes and/or damage either the within the motor cortex or along the pyramidal tracts.

In contrast to our increased stimulation threshold results, an ICMS study of rat forelimb maps after transient MCAO also reported the occurrence of small or nonexistent maps after stroke, yet currents up to 200 μA failed to elicit additional motor responses (41). This difference may be due to a longer occlusion duration leading to larger infarcts in the forelimb study. There were several rats in our study in which increased stimulation thresholds also failed to elicit a motor response, many of which had larger infarcts.

### Influence of infarct size on maps

We found that the size of the jaw activating region in the intact hemisphere was positively correlated with the size of the infarct, with larger infarcts associated with larger contralesional jaw activating regions (Figure 7). However, we did not find a similar relationship for tongue activation. This difference may be attributed to the pattern of infarction occurring after MCA occlusion in this model. Damage appears to be more centered on the tongue activating sensorimotor cortical areas, with only larger strokes advancing into the jaw activating cortical regions (Figure 2B). Therefore, the extent of damage to jaw activating areas is more likely to vary with the size of the stroke. It should also be considered that the smallest strokes were subcortical; one third of the strokes were subcortical with the rest a mix of cortical and subcortical—similar to the clinical stroke population (34, 78). Thus, in many cases, portions of the motor cortex that are not infarcted may fail to activate the jaw or tongue muscles because white matter damage has negatively affected connectivity of the cortex to the brainstem motor neurons.

## Conclusions and future work

This study found that old age and stroke affected swallowing metrics in a rat model. Further, we found differential neuroplasticity of jaw and tongue activation after stroke; only the jaw muscles were found to be activated by an increased volume of the sensorimotor cortex of the intact hemisphere, whereas significant differences in tongue activating cortical volume was limited to the infarcted hemisphere which was both significantly reduced with stroke and strongly associated swallowing function. This work also demonstrated that it is feasible to use aged F344 × BN rats for mechanistic studies of post stroke dysphagia. In this study we focused on the natural course of recovery after stroke, but clinically based protocols for therapies such as tongue exercise (22, 79–81) and neuromuscular electrical stimulation (82–84) are well established for this rodent model and future work should expand to focus on rehabilitation.

## Data availability statement

The raw data supporting the conclusions of this article will be made available by the authors, without undue reservation.

## Ethics statement

The animal study was approved by the University of Wisconsin School of Medicine and Public Health Animal Care and Use Committee. The study was conducted in accordance with the local legislation and institutional requirements.

## Author contributions

MC: Conceptualization, Formal analysis, Funding acquisition, Investigation, Methodology, Visualization, Writing – original draft, Writing – review & editing. NC: Conceptualization, Methodology, Funding acquisition, Writing – review & editing.
